# Hsp70 and Hsp90 Elaborately Regulate RNAi Efficiency in *Plutella xylostella*

**DOI:** 10.3390/ijms242216167

**Published:** 2023-11-10

**Authors:** Sujie Lin, Jie Yang, Weiqing Wang, Pengrong Huang, Muhammad Asad, Guang Yang

**Affiliations:** 1 State Key Laboratory of Ecological Pest Control for Fujian and Taiwan Crops, Institute of Applied Ecology, Fujian Agriculture and Forestry University, Fuzhou 350002, China; 2 Joint International Research Laboratory of Ecological Pest Control, Ministry of Education, Fuzhou 350002, China; 3 Key Laboratory of Integrated Pest Management for Fujian-Taiwan Crops, Ministry of Agriculture, Fuzhou 350002, China; 4 Key Laboratory of Green Pest Control (Fujian Agriculture and Forestry University), Fujian Province University, Fuzhou 350002, China

**Keywords:** Hsp70, Hsp90, RNA interference, *Plutella xylostella*

## Abstract

Heat-shock proteins (HSPs) serve as molecular chaperones in the RNA interference (RNAi) pathway of eukaryotic organisms. In model organisms, Hsp70 and Hsp90 facilitate the folding and remodeling of the client protein Argonaute (Ago). However, the specific function of HSPs in the RNAi pathway of *Plutella xylostella* (L.) (Lepidoptera: Plutellidae) remains unknown. In this study, we identified and analyzed the coding sequences of *PxHsc70-4* and *PxHsp83* (also known as *PxHsp90*). Both PxHsc70-4 and PxHsp83 exhibited three conserved domains that covered a massive portion of their respective regions. The knockdown or inhibition of *PxHsc70-4* and *PxHsp83* in vitro resulted in a significant increase in the gene expression of the dsRNA-silenced reporter gene *PxmRPS18*, leading to a decrease in its RNAi efficiency. Interestingly, the overexpression of PxHsc70-4 and PxHsp83 in DBM, Sf9, and S2 cells resulted in an increase in the bioluminescent activity of dsRNA-silenced luciferase, indicating a decrease in its RNAi efficiency via the overexpression of Hsp70/Hsp90. Furthermore, the inhibition of *PxHsc70-4* and *PxHsp83* in vivo resulted in a significant increase in the gene expression of *PxmRPS18*. These findings demonstrated the essential involvement of a specific quantity of Hsc70-4 and Hsp83 in the siRNA pathway in *P. xylostella*. Our study offers novel insights into the roles played by HSPs in the siRNA pathway in lepidopteran insects.

## 1. Introduction

The heat-shock proteins (HSPs) are a group of conserved proteins synthesized by cells in response to various stress conditions. HSPs, which function as molecular chaperones, are ubiquitous in cells and assist other proteins in coping with stress-induced denaturation. Among these, Hsp70 and Hsp90 are two highly conserved ATP-consuming chaperone families [1]. These proteins, in conjunction with other protein factors, facilitate the folding, remodeling, and maturation of numerous client proteins, although HSPs also possess independent chaperone activities [2]. For instance, the chaperone machines Hsp70 and Hsp90 play a complementary role in regulating the functionality of the tumor suppressor protein p53 by continuously modifying its structure and effectively maintaining a balance between its flexibility and stability [3,4]. The intricate molecular mechanisms underlying the chaperone cycle have been elucidated by the Agard laboratory [5,6]. The client protein glucocorticoid receptor (GR) is found to be partially unfolded and recognized by the extended binding pocket formed by Hsp90, Hsp70, and Hop [5]. As the GR ligand-binding domain passes through the Hsp90 lumen, it undergoes a conformational change and returns to a folded state, and Hsp90 directly determines the client-protein-specific folding outcome [6].

In insects, the investigation of HSPs has primarily centered on their various functions in physiological development and response to environmental stressors [7], such as heat [8], cold [9], pathogens [10], anoxia [11] and UV [12]. Meanwhile, HSPs have been selected as candidate target genes for RNAi-based pest management [13,14,15]. In *P. xylostella*, research related to HSPs has mainly focused on thermal tolerance, insecticide resistance and other abiotic stresses [16,17,18]. So far, studies of insect HSPs focusing on RNAi mechanisms have been conducted primarily on *Drosophila melanogaster* (Diptera: Drosophilidae) [19,20,21]. Within the RNA interference (RNAi) pathway of *D. melanogaster*, the Hsp70/Hsp90 chaperone machinery comprises Hsc70-4, a member of the heat-shock cognate protein 70, and Hsp83 (Hsp90 homolog). These chaperones play a specific role in facilitating the reception of the small RNA duplex from the RNA-induced silencing complex (RISC) by Ago1 and Ago2 [19,22]. The reconstitution of *D. melanogaster* RISC assembly involves multiple proteins, in which Hsp70 and Hsp90 extend the dwell time of the Dicer–siRNA complex on Ago2 [20]. Specifically, Hsc70-4 populates the open form of Ago2, and Hsp83 captures and stabilizes the active form [21]. Additionally, the Hsp70/Hsp90 complex is involved in piRNA biogenesis by triggering stress-induced transposable element (TE) activation in the reproductive system of flies [23]. In human and plant cells, Hsp90 aids in the assembly of RISC and accompanies the Argonaute protein prior to RNA binding [24,25]. Similar functions have been observed in the budding yeast *Saccharomyces castellii* (Saccharomycetales: Saccharomycetaceae) and the protozoan *Tetrahymena thermophila* (Hymenostomatida: Tetrahymenidae), where Hsp90 is involved in regulating the Argonaute activities [26,27].

The diamondback moth (DBM), *P. xylostella*, is a notorious lepidopteran pest that feeds on cruciferous crops, leading to huge economic losses annually [28]. Given its notable resistance to pesticides [29], the utilization of RNAi-based control methods presents a potential alternative to chemical insecticides [30]. RNAi-based products can specifically silence lethal genes to cause insect mortality in an environmentally friendly manner [31,32]. On the other hand, RNAi efficiency is variable in *P. xylostella*, where RNAi works inefficiently through dsRNA microinjection, dsRNA-producing engineer bacteria and transgenic plants [33,34,35,36]. Therefore, it is imperative to comprehensively understand the RNAi pathway in lepidopteran insects.

In this study, we first identified and characterized the molecular chaperones PxHsc70-4 and PxHsp83 (named after homologous sequences of *D. melanogaster*), after which we analyzed their expression profiles. We then assessed their impact on RNAi efficiency through in vitro transfection experiments using RNAi-of-RNAi and RNAi-of-inhibition strategies. Furthermore, we overexpressed PxHsc70-4 and PxHsp83 in insect cells to uncover their suppressive effect on RNAi efficiency. Finally, in vivo injection experiments were conducted to evaluate the effects of PxHSP knockdown or inhibition on the RNAi efficiency of the reporter gene.

## 2. Results

### 2.1. Sequence Characterization of Hsc70-4 and Hsp83 in P. xylostella

**Figure 1:**
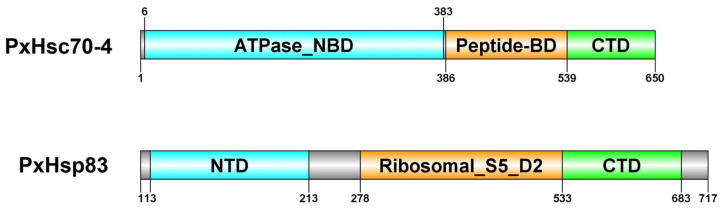
Schematic diagram of the domains of PxHsc70-4 and PxHsp83. Gray, intron; cyan, ATPase nucleotide-binding domain (ATPase_NBD), N-terminal domain (NTD); orange: peptide-binding domain (peptide-BD), ribosomal protein S5 domain 2-type fold (Ribosomal_S5_D2); green: C-terminal domain (CTD). Numbers represent the amino acid sites.

**Figure 2:**
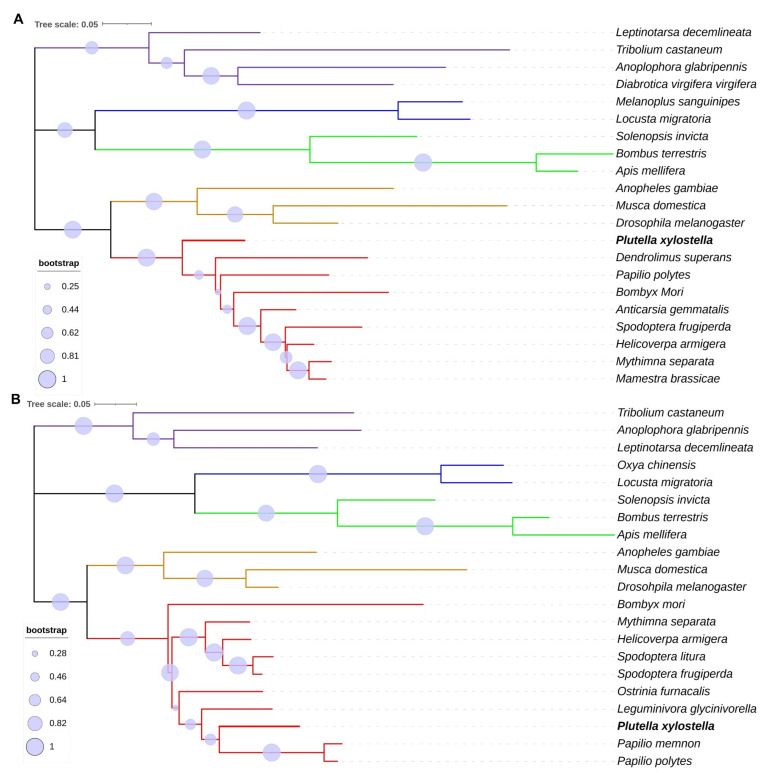
Phylogenetic analysis of Hsc70-4 and Hsp83 across 21 insect species. (**A**) Hsc70-4 genes were from four Coleoptera (purple), two Orthoptera (blue), three Hymenoptera (green), three Diptera (orange) and ten Lepidoptera (red). (**B**) Hsp83 genes were from three Coleoptera, two Orthoptera, three Hymenoptera, three Diptera and ten Lepidoptera. The maximum likelihood method was used to construct the phylogenetic tree with 1000 bootstrap replications, and the tree scale was provided.

The *PxHsc70-4* gene consists of a 1953-bp open reading frame (ORF) that is composed of two exons and codes for a protein consisting of 650 amino acids (aa). This protein has a predicted molecular weight of 71.18 kDa and an isoelectric point (pI) of 5.32 (Table 1). The *PxHsp83* gene spans a length of 2154 bp and contains a single exon. It encodes a protein consisting of 717 aa, with a molecular weight of 82.46 kDa and a pI of 4.98 (Table 1). PxHsc70-4 was predicted to be localized in the nucleus, while PxHsp83 was predicted to be localized in three organelles (Table 1). The two proteins exhibited three distinct conserved domains: the ATPase nucleotide-binding domain (ATPase_NBD) or N-terminal domain (NTD), the peptide-binding domain (peptide-BD) or ribosomal protein S5 domain 2-type fold (Ribosomal_S5_D2), and the C-terminal domain (CTD), which encompassed the majority of the regions (Figure 1). In the phylogenetic trees, a total of 21 species were classified into five groups with the same order, respectively (Figure 2). PxHsc70-4 diverged earlier with lower evolutionary variability (Figure 2A). The genetic distances of Hsc70-4 between *P. xylostella* and other lepidopteran insects indicate a relatively distant relationship (Figure 2A). A compared pattern was observed in the phylogenetic tree of Hsp83, where the lepidopteran group exhibited the minimal evolutionary variability of Hsp83, except for *B. mori* (Figure 2B). Furthermore, both Hsp83 and Hsc70-4 in Lepidoptera displayed a close relationship with those in Diptera (Figure 2B). Overall, Hsc70-4 and Hsp83 exhibited a similar trend of genetic distance in different insect species and were more conserved in Lepidoptera.

### 2.2. Expression Profiles of HSPs

**Figure 3:**
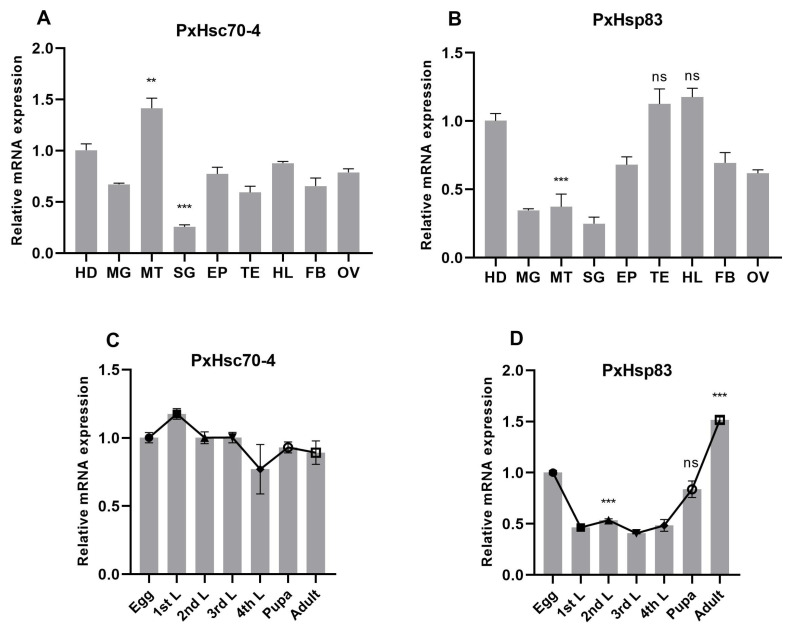
Expression profiles of *PxHsc70-4* and *PxHsp83* in different tissues (**A**,**B**) and developmental stages (**C**,**D**). HD: head, MG: midgut, MT: Malpighian tubule, SG: silk gland, EP: epidermis, TE: testis, HL: hemolymph, FB: fat body, OV: ovary, L: larvae that contain four stages. *RPL32* was used to normalize the mRNA expression levels. Each experiment was repeated three times (mean ± SE, *n* = 3). Statistical significance was determined via one-way ANOVA with Tukey’s test (ns, no significance; **, *p* < 0.01; ***, *p* < 0.001).

The tissue-specific expression profiles demonstrated that *PxHsc70-4* exhibited the highest expression in the Malpighian tubule, the lowest level in the silk gland, and moderate levels in other tissues (Figure 3A). Conversely, *PxHsp83* displayed high expression levels in the hemolymph, testis and head, while exhibiting low levels in the midgut, Malpighian tubule and silk gland (Figure 3B). The developmental expression profiles revealed that *PxHsc70-4* maintained a stable expression throughout all stages (Figure 3C), whereas *PxHsp83* exhibited heightened expression during the egg and adult stages, with a significant increase observed after the pupal stage (Figure 3D).

### 2.3. Knockdown of HSPs In Vitro

**Figure 4:**
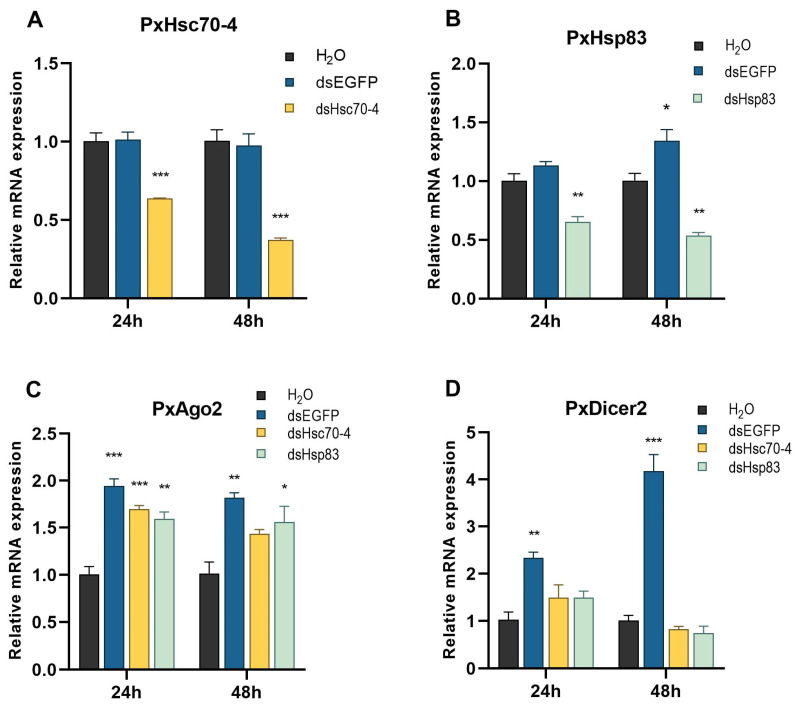
Effect of HSP knockdown on the expression of RNAi genes in vitro. (**A**) Relative mRNA expression of *PxHsc70-4* after dsHsc70-4 transfection in 24–48 h. (**B**) Relative mRNA expression of *PxHsp83* after dsHsp83 transfection in 24–48 h. (**C**,**D**) Relative mRNA expression of *PxAgo2* and *PxDicer2* after dsHSP transfection in 24–48 h. DsEGFP and H_2_O were used as a negative control and a blank control, respectively. Each experiment was repeated three times (mean ± SE, *n* = 3). Statistical significance was determined via one-way ANOVA with Fisher’s LSD (*, *p* < 0.05; **, *p* < 0.01; ***, *p* < 0.001) and Tukey’s test (*, *p* < 0.05; **, *p* < 0.01; ***, *p* < 0.001), respectively.

During various time intervals, the mRNA expression of *PxHsc70-4* exhibited significant suppression within the 12–72 h range (Figure S1A), while the suppression of *PxHsp83* was observed within the 24–48 h range (Figure S1B). Consequently, the 24–48 h period, with optimal knockdown efficiency, was selected to assess alterations in the mRNA expression of core RNAi genes. The expression of *PxHsc70-4* in the dsHsc70-4 treatment was reduced by approximately 41.01% and 52.37% compared to the dsEGFP treatment at 24 h and 48 h, respectively (Figure 4A). Similarly, the expression of *PxHsp83* was reduced by approximately 38.53% and 44.86% (Figure 4B). For the core RNAi genes, the expression levels of *PxAgo2* and *PxDicer2* were evaluated in response to four treatments at 24 h and 48 h. Compared to the control treatment (H_2_O), the expression of *PxAgo2* was significantly increased in all treatments involving dsRNAs (dsHsc70-4, dsHsp83 and dsEGFP) (Figure 4C). Additionally, the expression of *PxDicer2* was found to be higher in the dsEGFP treatment compared to the other three treatments (dsHsc70-4, dsHsp83 and H_2_O), although no significant differences were observed among the latter (Figure 4D). These findings suggested that *Hsc70-4* and *Hsp83* could be effectively silenced. Furthermore, they suggested that the core RNAi genes could be induced by dsRNAs in DBM cells, while the presence of dsHSPs hampered the dsRNA-induced expression of *PxDicer2*.

### 2.4. Effect of HSP Knockdown on RNAi Efficiency In Vitro

**Figure 5:**
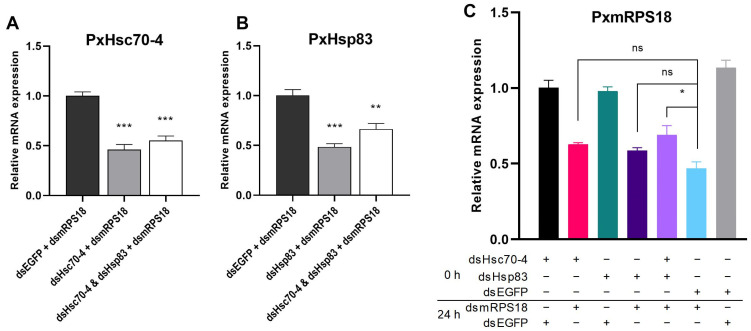
Effect of HSP knockdown on the RNAi efficiency of *PxmRPS18* in vitro. Relative mRNA expression of *PxHsc70-4* (**A**), *PxHsp83* (**B**) and *PxmRPS18* (**C**) after dsHSP and dsmRPS18 transfection in turn. DsEGFP was used as a control. Each experiment was repeated three times (mean ± SE, *n* = 3). Statistical significance was determined via one-way ANOVA with Tukey’s test (ns, no significance; *, *p* < 0.05; **, *p* < 0.01; ***, *p* < 0.001).

The reporter gene *PxmRPS18*, which is a member of the mitochondrial ribosomal protein family, was selected for efficient RNAi-mediated knockdown. The expressions of HSPs were assessed after RNAi-of-RNAi, revealing a substantial decrease in the dsHSP-transfected groups (Figure 5A,B). The co-transfection of dsHsc70-4 and dsHsp83 with a halved dosage each was as highly effective as separate transfection in silencing the expressions of Hsp70 and Hsp90, respectively (Figure 5A,B). The experimental design involved seven treatments, and the expression of *PxmRPS18* was measured at 24 h after the second transfection. Significant reductions in *PxmRPS18* expression were observed in the dsmRPS18 treatments compared to the dsEGFP treatments (Figure 5C). The expression of *mRPS18* in the treatments involving dsHsc70-4 + dsmRPS18 and dsHsp83 + dsmRPS18 did not show any significant differences compared to the treatment involving dsEGFP + dsmRPS18 (Figure 5C). However, a significant increase was observed in the treatment involving dsHsc70-4 + dsHsp83 + dsmRPS18 (Figure 5C), indicating that Hsc70-4 and Hsp83 might collaborate in the RNAi process of *P. xylostella* in vitro.

### 2.5. Effect of HSP Inhibition on RNAi Efficiency In Vitro

**Figure 6:**
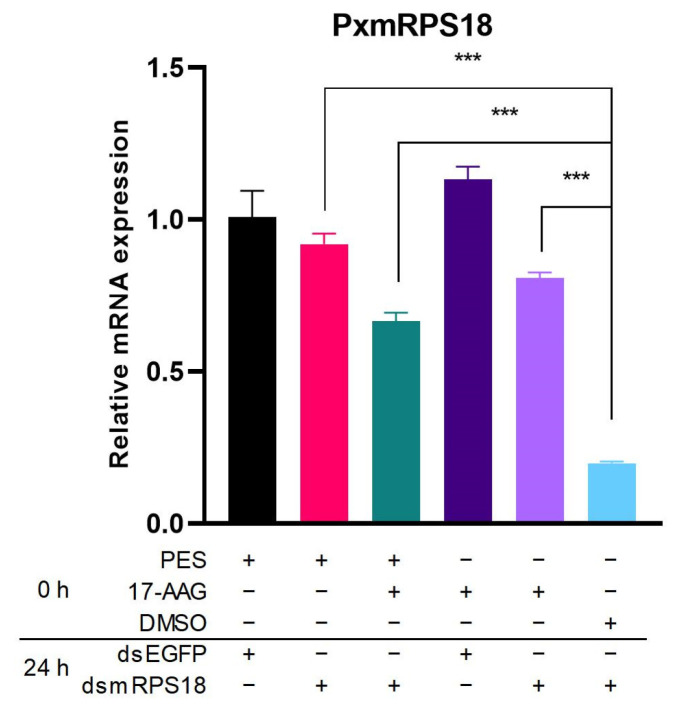
Effect of HSP inhibition on RNAi efficiency in vitro. Effect of HSP inhibitor transfection on the mRNA expression of *PxmRPS18*. DMSO and dsEGFP were, respectively, used as controls of the inhibitor and dsRNA. Each experiment was repeated three times (mean ± SE, *n* = 3). Statistical significance was determined via one-way ANOVA with Tukey’s test (***, *p* < 0.001).

After the addition of HSP inhibitors, PES or 17-AAG unexpectedly led to a significant increase in the mRNA expression of *PxHsc70-4* or *PxHsp83* (Figure S3). In RNAi-of-inhibition assays, the addition of inhibitors led to significant increases in *mPRS18* expression (Figure 6). These finding suggested that the inhibition of HSPs impeded the mRNA expression of *mRPS18*, and that the RNAi efficiency of *mRPS18* was positively regulated by HSPs.

### 2.6. Effect of HSP Overexpression on RNAi Efficiency In Vitro

**Figure 7:**
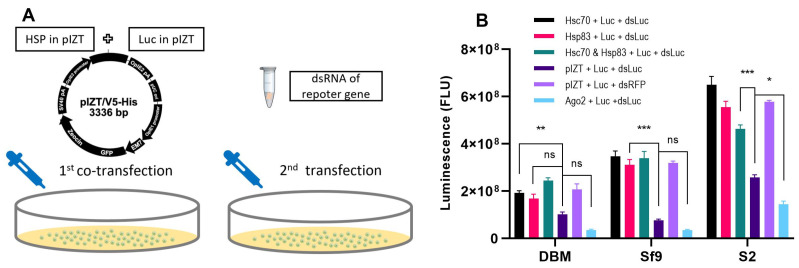
Effect of PxHSP overexpression on the RNAi efficiency of Luc in DBM, Sf9 and S2 cells. (**A**) Schematic diagram of RNAi-of-overexpression, with 1st co-transfection of pIZT constructs expressing Hsp70/Hsp90 and Luc, and 2nd transfection of dsmRPS18. (**B**) Effect of pIZT-HSP and pIZT-Luc co-transfection followed by Luc knockdown on the luciferase activity. The pIZT vector and pIZT-Ago2 were used as a negative control and a positive control, respectively. Each experiment was repeated three times (mean ± SE, *n* = 3). Statistical significance was determined via one-way ANOVA with Tukey’s test (ns, no significance; *, *p* < 0.05; **, *p* < 0.01; ***, *p* < 0.001).

Hsc70-4, Hsp83 and luciferase were individually inserted into the pIZT/V5-His vector (Figure 7A). The pIZT–Hsc70-4 and pIZT–Hsp83 vectors were expressed using the OpIE2 promoter in DBM, Sf9 and S2 cells (Figure S2). Additionally, the core RNAi gene *PxAgo2* was inserted into the pIZT vector and served as a positive control. In comparison to the control group transfected with the empty pIZT vector, the overexpression of PxAgo2 resulted in a significant reduction in luciferase activity in S2 cells exposed to dsLuc. Similarly, a modest decrease in luciferase activity was observed in DBM and Sf9 cells (Figure 7B), indicating a higher RNAi efficiency following the overexpression of PxAgo2. Surprisingly, the upregulation of PxHsc70 or PxHsp83 resulted in a significant increase in luciferase activity in DBM cells treated with dsLuc, as well as in Sf9 and S2 cells through the heterologous expression of PxHSPs (Figure 7B). These findings indicated that the overexpression of Hsc70-4 or Hsp83 hampered the effectiveness of RNAi in insect cells, suggesting that an excessive abundance of Hsp70/Hsp90 might have a counterproductive impact. It is therefore crucial for cells to maintain an optimal level of HSPs to ensure efficient RNAi.

### 2.7. Effect of HSP Inhibition on RNAi Efficiency In Vivo

**Figure 8:**
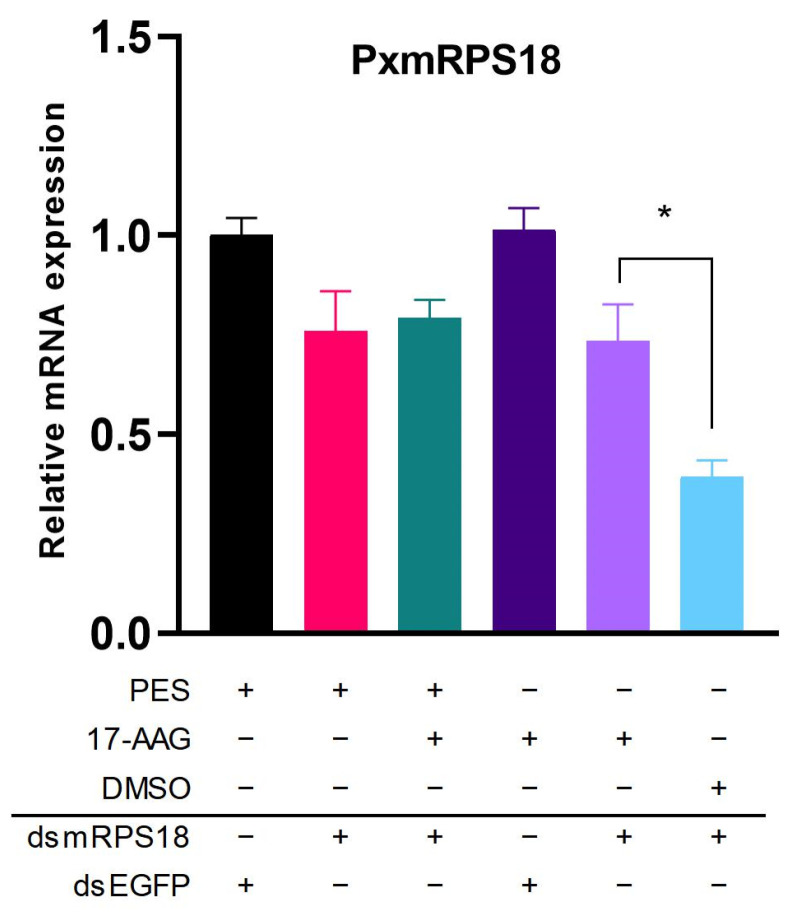
Effect of HSP inhibition on the RNAi efficiency of *PxmRPS18* in vivo. Effect of PES/17-AAG and dsmRPS18 co-injection on the mRNA expression of *PxmRPS18* in vivo. DsEGFP and DMSO were used as negative controls. Each experiment was repeated three times (mean ± SE, *n* = 3). Statistical significance was determined via one-way ANOVA with Tukey’s test (*, *p* < 0.05).

In the in vivo RNAi-of-RNAi assay, the co-injection of dsHSP and dsmRPS18 into the bodies of 4th-instar larvae resulted in significantly low levels of mRNA expression for *PxHsc70-4* and *PxHsp83* (Figure S4A,B). However, when the dsRNA-mediated knockdown of *mRPS18* was performed, there were no significant differences observed between treatments with or without dsHSPs, as anticipated (Figure S4C). Substituting dsHSPs with the HSP inhibitors PES and 17-AAG led to a significant increase in the RNAi efficiency of *mRPS18* (Figure 8). These data demonstrated that the in vivo administration of dsHSPs did not have a significant impact on the RNAi efficiency. Conversely, injections of Hsp70/Hsp90 inhibitors exhibited a considerable potential to decrease the RNAi efficiency in vivo. These findings indicated a poor RNAi efficiency in *P. xylostella* in vivo and highlighted the essential role of Hsp70/Hsp90 in the RNAi process in this species.

## 3. Discussion

Hsc70-4 and Hsp83 functioning as molecular chaperones are integral to maintaining protein homeostasis and are involved in nearly all cellular processes. Here, we identified and analyzed *Hsc70-4* and *Hsp83* genes in *P. xylostella*. PxHsc70-4 was predicted to localize in the nucleus, potentially due to its association with a network of cytoplasmic fibers concentrated around the nucleus [37]. Similarly, PxHsp83 was predicted to be localized in three organelles, resembling the localization pattern observed in the fly Hsp83 [38,39,40]. Both PxHsc70-4 and PxHsp83 possessed three conserved domains. These conserved domains cover a significant portion of the sequences, which align with the typical domains found in Hsp70 and Hsp90 [41,42], indicating their highly conserved characteristics.

A significantly higher expression of *PxHsp83* at 48 h in the dsEGFP group suggested the potential enhancing effect of exogenous dsRNA on genes relative to the siRNA pathway. The addition of dsRNA also enhanced the expressions of core RNAi genes to varying extents. This finding aligns with previous research showing that injection with dsRNA in *Manduca sexta* (Lepidoptera: Sphingidae) induces a specific upregulation of *Dicer2* [43]. It is likely that the transfection of dsEGFP promotes, rather than hinders, the RNAi effect by augmenting *Dicer2* expression. Similar observations have been made in virus-infected or abiotic-stress-treated insects [44,45].

Only the co-transfection of dsHsc70-4 and dsHsp83 could cause a reduction in the RNAi efficiency of *mRPS18*, which accorded with their complementary functions via an intricate interplay and cooperation when regulating the p53 activity [3,4]. This phenomenon was reminiscent of the synergistic effects observed in two distinct G proteins, which together activate the signaling priming mechanism of G-protein-coupled receptor [46], as well as the redundant/complementary effects showing that the double knockout of *PxABCC2* and *PxABCC3* in *P. xylostella* confers a higher level of Cry1Ac resistance than separate knockout [47].

When two small molecule inhibitors were employed to inhibit the activity of HSP proteins, the mRNA expressions of Hsp70 and Hsp90 were significantly increased but not decreased. This observed increase could possibly be explained by the mechanisms by which the HSP inhibitions prompted extensive mRNA transcription for preserving intracellular homeostasis or responding to certain feedback mechanisms. This dynamic association between mRNA and protein levels was influenced by variations in the half-life or the physiological environment inside the cells [48]. Interestingly, in contrast to the observed findings with dsHSP transfection, the inhibitory impact on the RNAi efficiency of the reporter gene via coaddition was less pronounced than their individual additions. This disparity might be due to the antagonistic effects of the two HSP inhibitors, as well as the reduction in PES dosage contributing to a weakening of their overall functionality. Nonetheless, our results indicated that either PES or 17-AAG could greatly inhibit the RNAi efficiency of the reporter gene, which was attributed to the mechanisms wherein PES interacts with Hsp70 by hindering its binding with client proteins [49] and 17-AAG effectively competes with ATP by tightly binding to the ATPase of Hsp90 [50]. These results agreed with the findings, in which PES or 17-AAG reduced the RISC activity for target cleavage, thus inhibiting the RNAi activity [19].

It was reported that the overexpression of the *Leptinotarsa decemlineata* (Coleoptera: Chrysomelidae) genes Ago1, Ago2b, Aub and Vha16 in RNAi refractive Sf9 cells improved RNAi efficiency [51]. This finding contrasts with the results from a previous study on HSPs, where Hsp70/Hsp90 did not appear to affect the luciferase activity because the monomeric protein functions immediately upon translation without post-translational modification [52]. Notably, the abundantly expressed Hsc70-4 and Hsp83 contribute to cellular homeostasis by buffering negative impacts under dynamic cellular environments [42]. Therefore, a possible explanation might be that cells require a precise quantity of HSPs to carry out their chaperone functions. The excessive presence of Hsp70/Hsp90 disrupted cellular homeostasis, thereby interfering with the RNAi process. This finding aligned with previous observations that demonstrate a consistent phenomenon arising from a precise regulation of miRNA and its target gene, whether through the overexpression or knockdown of miRNA [53,54]. It is noteworthy that there is a scarcity of reported functional validation experiments concerning the overexpression of HSPs. Our findings provide support for the elaborate regulation mode in cells. Recently, the utilization of Hsp70 and Hsp90 proteins was observed in the safeguarding of Cry1A protoxin and the augmentation of its toxicity when fed as a recombinant protein to *P. xylostella* larvae [55,56]. Despite the unclear residual activity impeded by protein degradation in guts, the in vivo oral delivery of recombinant Hsp70/Hsp90 proteins with dsRNA merits investigation in future.

In our experiments, the effect of HSP inhibitors on RNAi efficiency was better than that of dsHSPs, which could be explained by the fact that the HSP inhibitors simultaneously suppressed the Hsp70 or Hsp90 isoforms, thereby preventing their compensatory expression from taking over the role of Hsc70-4 or Hsp83 [57]. The utilization of HSP inhibitors but not dsHSPs was capable of impeding the RNAi efficiency of the reporter gene in vivo. Combined with the results in vitro, we suggested that Hsp70 and Hsp90 were required for efficient RNAi processes. Additionally, whether mediated by inhibitors or dsRNA, the RNAi efficiency in vitro was generally higher than in vivo. A RNAi-of-RNAi experiment implemented in the RNAi-resistant insect *Ostrinia furnacalis* (Lepidoptera: Crambidae) ex vivo using a tissue culture technique also served as an example [58]. These cases demonstrated that the uptake of exogenous dsRNA into cells partly causes resistance to RNAi. Further studies should be undertaken to investigate this perspective.

The CRISPR/Cas9 technology proves particularly valuable in investigating factors associated with the RNAi pathway of RNAi-resistant insects [59,60,61]. Meanwhile, the CRISPR-mediated Hsp70/Hsp90 knockout was hard to perform as they are essential for pest survival. For example, they were utilized as highly efficient targets for killing agricultural pests through robust RNAi [14,15]. Hence, an in vitro active reaction system of RNAi constructed by recombinant proteins deserves to be developed for verifying the gene functions.

It is noteworthy that insects may develop resistance to RNAi-based products via the mutation of target genes or RNAi core machinery genes [62]. Herein, HSPs serve as both target genes and RNAi-related genes. Target sites in the highly conserved Hsp70/Hsp90 must be differentiated from their respective isoforms for the specific knockdown of target genes. On the other hand, whether other isoforms take over the role of HSPs in the RNAi process needs to be explored. In terms of RNAi-related genes, because the resistance of *P. xylostella* to chemical and microbial pesticides is highly correlated with the significant upregulation or downregulation of HSPs [17,18,63], it is suggested that HSPs may participate in the development of resistance to insecticides in *P. xylostella*, which raises the possibility that there is a correlation between HSPs and RNAi resistance in *P. xylostella*.

## 4. Materials and Methods

### 4.1. Insects and Cells

The susceptible DBM strain (FZ-S) was initially collected in the farmland of Fuzhou (Fujian, China) in 2004 [64] and maintained in the Institute of Applied Ecology, Fujian Agriculture and Forestry University. The *P. xylostella* larvae reared on potted radish seedlings were maintained in a greenhouse at 25 ± 0.5 °C and 60 ± 5% relative humidity, with a 16 h/8 h (light/dark) cycle. The DBM cells isolated from embryos of the DBM strain Geneva 88 [65] were grown in Grace’s insect medium (Gibco, Carlsbad, CA, USA) supplemented with 10% FBS (Gibco, Carlsbad, CA, USA). The Sf9 cell line derived from *Spodoptera frugiperda* (Lepidoptera: Noctuidae) ovaries was purchased from Thermo Scientific and cultured with Sf-9000 III SFM (Gibco, Carlsbad, CA, USA) containing 5% FBS. The S2 cell line derived from *D. melanogaster* embryos (20–24 h old) was cultured in Grace’s insect medium supplemented with 10% FBS. All the cells were attached in T-25 flasks at 27 °C.

### 4.2. Gene Cloning and Analysis

The total RNA isolated from the 4th-instar larvae using the Eastep Super Total RNA Extraction Kit (Promega, Madison, WI, USA) was reversed to the first-strand cDNA using the GoScript Reverse Transcription System (Promega, Madison, WI, USA). The *PxHsc70-4* reference sequence was acquired from the NCBI (https://www.ncbi.nlm.nih.gov/ accessed on 20 June 2018) via blasting with the Hsc70-4 CDS sequence of *D. melanogaster* (FlyBase ID: FBgn0266599), and the *PxHsp83* (gene ID: Px010238) was downloaded from the DBM-DB database (http://iae.fafu.edu.cn/DBM/, accessed on 19 June 2018) [66] and the sequence was confirmed using the NCBI BLAST. The gene sequences were amplified via PCR using the Phanta Max Super-Fidelity DNA Polymerase (Vazyme, Nanjing, China) with the primers shown in Table S1. Then, the purified products were inserted into the pJET1.2 vector (Thermo Fisher Scientific, Waltham, MA, USA) for sequencing.

The conserved domain family was identified using the NCBI CD search tool (https://www.ncbi.nlm.nih.gov/Structure/cdd/wrpsb.cgi, accessed on 3 April 2020). The subcellular localization of proteins was predicted using Ceel-Ploc-2 (http://www.csbio.sjtu.edu.cn/bioinf/Cell-PLoc-2/, accessed on 5 April 2020). The isoelectric points (PIs) and molecular weights (MWs) were estimated using ExPASy (http://web.expasy.org/compute_pi, accessed on 5 April 2020). The putative domains were predicted with Interpro (https://www.ebi.ac.uk/interpro/, accessed on 5 April 2020) and visualized using GOD2.0 (http://dog.biocuckoo.org/, accessed on 18 August 2021). The phylogenetic trees of Hsc70-4 and Hsp83 were individually constructed using MEGA 11 with DNA sequences from 21 insect species (Table S2). The maximum likelihood method with 1000 replications was used, and the images were drawn using iTOL (http://itol.embl.de/, accessed on 12 May 2022).

### 4.3. qRT-PCR Analysis

The total RNA was isolated from the DBM cells, whole body or tissues of *P. xylostella* larvae by using the Eastep Super Total RNA Extraction Kit (Promega, Madison, WI, USA). The first-strand cDNA was synthesized with Oligo (dT)_15_ primers by using the GoScrip Reverse Transcription System (Promega, Madison, WI, USA). The qRT-PCR reactions with specific primers (Table S1) were performed in the CFX96 Real-Time PCR Detection System (Bio-Rad, Hercules, CA, USA) using the Eastep qPCR Master Mix (Promega, Madison, WI, USA). Each reaction was performed with three independent replications and three technical repeats. For relative quantification analysis, the 2^−∆∆ct^ method [67] was applied to calculate the expression of target genes. The data regarding the threshold cycle values were normalized to the expression level of ribosomal protein L32 (*RPL32*).

### 4.4. Expression Pattern of Hsp70/Hsp90

For tissue-specific analysis, the head, midgut, Malpighian tubule, silk gland, epidermis, testis, hemolymph, fat body and ovary from 4th-instar larvae were dissected under the stereomicroscope and were separately kept in RNA Stabilization Reagent (Qiagen, Venlo, The Netherlands). For stage-specific analysis, the eggs, larvae (1st L–4th L), pupae, and adults were individually pooled. RNA isolation, cDNA synthesis and qRT-PCR were performed as described above.

### 4.5. DsRNA Synthesis

The first-strand cDNA was used as a template for the PCR amplification of the DNA fragments of the target genes, and EGFP/RFP/Luc constructs were used for the amplification of the DNA fragments of the control or reporter genes. The PCR primers contained the T7 promoters flanking both sides of the amplicon (Table S1). The purified PCR products were used as templates for in vitro transcription at 37 °C using the T7 RiboMAX Express RNAi System (Promega, Madison, WI, USA). The dsRNA of EGFP or RFP was used as a control and synthesized under the same conditions. After the purification of dsRNA, the RNA concentration was determined using the NanoDrop2000 spectrophotometer (Thermo Fisher Scientific, Waltham, MA, USA) and using by 1% (*w*/*v*) agarose gel electrophoresis.

### 4.6. RNAi-of-RNAi Assay In Vitro and In Vivo

To prime the process of RNAi-of-RNAi assays, the RNAi efficiency of *Hsc70-4* and *Hsp83* knockdown was estimated in advance. In total, 2 μg of dsHsc70-4 or dsHsp83 mixed with 4 μL of the cellfectin II reagent (Invitrogen, Waltham, MA, USA) was transfected into 12-well plates. DBM cells were harvested to determine the HSP knockdown via qRT-PCR at 12, 24, 48 and 72 h. Then, the mRNA expressions of *Hsc70-4* and *Hsp83,* using three treatments, and the core RNAi genes *Ago2* and *Dicer2*, using four treatments, were determined at proper time periods via qRT-PCR. DsEGFP and nuclease-free water were used as a negative control and a blank control, respectively. For the in vitro assay, *mRPS18* was used as an effective reporter gene for monitoring RNAi efficiency. DsRNA transfection was carried out twice. First, 2 μg of dsHsc70-4 or dsHsp83 mixed with 4 μL of cellfectin II was added to plates; then, 2 μg of dsmRPS18 was added in the same plates using cellfectin II at 24 h post transfection. Then, 24 h later, DBM cells were harvested, and the mRNA expression of *PxmRPS18* was determined via qRT-PCR. DsEGFP was used as a negative control twice. For the in vivo assay, 0.3 μg of dsHSP and dsmRPS18 was mixed and co-injected into the 4th-instar larvae at the position of the intersegmental membrane in the abdomen using the microinjector Nanoject III (Drummond Scientific, Birmingham, AL, USA). The injected larvae were collected at 48 h post injection, and qRT-PCR was applied to determine the mRNA expression of *PxmRPS18*.

### 4.7. RNAi-of-Inhibition Assay In Vitro and In Vivo

The pharmacological inhibitors (MedChemExpress, Monmouth Junction, NJ, USA) 2-phenylethynesulfonamide (PES) for Hsc70-4 and tanespimycin (17-AAG) for Hsp83 were dissolved in dimethyl sulfoxide (DMSO) and added into 12-well plates at final concentrations of 20 μM and 1 μM, respectively. DBM cells were harvested to isolate the total mRNA. The mRNA expressions of HSPs were determined via qRT-PCR. For the in vitro assay, the inhibitors and dsRNA were transfected into cells sequentially. Firstly, PES or 17-AAG was added to the plates, and the cells were incubated for 24 h. Then, dsmRPS18 combined with cellfectin II was added to each plate. The cells were harvested, and the mRNA expression of *PxmRPS18* was determined via qRT-PCR. DMSO was used as a blank control in the first transfection and dsEGFP was used as a negative control in the second transfection. For the in vivo assay, 100 nL of PES (2 mM) or 17-AAG (100 μM) mixed with 0.3 μg of dsmRPS18 was simultaneously co-injected into the 4th-instar larvae. DMSO and dsEGFP were used as controls for the inhibitors and dsmRPS18, respectively. Six different treatments were collected and analyzed via qRT-PCR in 48 h.

### 4.8. Overexpression and Functional Verification in Insect Cells

The coding sequences of Hsc70-4, Hsp83, Ago2 and Luc were separately cloned into the pIZT/V5-His expression vectors by using the restriction enzyme sites of EoRI and KpnI. DBM, Sf9 and S2 cells were prepared at 60–70% confluency for transfection. Then, 0.5 μg of the pIZT vectors (Luc construct + expression construct) mixed with 1 μL of cellfectin II was transfected into 48-well plates. Then, 0.5 μg of dsLuc mixed with 1 μL of cellfectin II was added to the medium at 24 h post transfection, followed by cell collection 24 h later. For the luciferase reporter assay, cells rinsed with 1× PBS were dispensed with 1× PLB lysis buffer (Promega, Madison, WI, USA) for 15 min. The lysate was transferred to 96-well plates. Then, 100 μL of LAR II was dispensed into each well to measure the luciferase activity by using the GloMax Navigator (Promega, Madison, WI, USA). Each treatment was independently performed three times with three technical replicates.

## 5. Conclusions

Our study highlighted that Hsc70-4 and Hsp83 play important roles in the siRNA pathway. The involvement of PxHsc70-4 and PxHsp83 in the siRNA-mediated RNAi process in *P. xylostella* was characterized by their precise regulation manner. Cells should accurately maintain a specific quantity of HSPs to ensure optimal RNAi efficiency. Our research provides novel insights into the relationship between Hsp70/Hsp90 and the RNAi mechanism in Lepidoptera.

## Figures and Tables

**Table 1:** Summary of *Hsp70/Hsp90* genes in *P. xylostella*.

Gene Name	Protein Length (aa)	Domain Family	Localization	Exon	PI	MW (kDa)	GenBank Accession Number
***PxHsc70-4***	650	PTZ00009	Nucleus	2	5.32	71.18	JN676213.1
***PxHsp83***	717	PTZ00272	Centrosome. Cytoplasm. Mitochondrion	1	4.98	82.46	NM_001309114.1

## Data Availability

All data generated or analyzed during this study are included in this published article (and its Supplementary Materials files).

## Supplementary Materials

The supporting information can be downloaded at: https://www.mdpi.com/article/10.3390/ijms242216167/s1.

## References

[1] Feder M.E., Hofmann G.E. (1999). Heat-Shock Proteins, Molecular Chaperones, and the Stress Response: Evolutionary and Ecological Physiology. Annu. Rev. Physiol..

[2] Genest O., Wickner S., Doyle S.M. (2019). Hsp90 and Hsp70 Chaperones: Collaborators in Protein Remodeling. J. Biol. Chem..

[3] Boysen M., Kityk R., Mayer M.P. (2019). Hsp70- and Hsp90-Mediated Regulation of the Conformation of P53 DNA Binding Domain and P53 Cancer Variants. Mol. Cell.

[4] Dahiya V., Agam G., Lawatscheck J., Rutz D.A., Lamb D.C., Buchner J. (2019). Coordinated Conformational Processing of the Tumor Suppressor Protein P53 by the Hsp70 and Hsp90 Chaperone Machineries. Mol. Cell.

[5] Wang R.Y.-R., Noddings C.M., Kirschke E., Myasnikov A.G., Johnson J.L., Agard D.A. (2022). Structure of Hsp90–Hsp70–Hop–GR Reveals the Hsp90 Client-Loading Mechanism. Nature.

[6] Noddings C.M., Wang R.Y.-R., Johnson J.L., Agard D.A. (2022). Structure of Hsp90–P23–GR Reveals the Hsp90 Client-Remodelling Mechanism. Nature.

[7] King A.M., MacRae T.H. (2015). Insect Heat Shock Proteins during Stress and Diapause. Annu. Rev. Entomol..

[8] Ritossa F. (1996). Discovery of the Heat Shock Response. Cell Stress Chaperones.

[9] Rinehart J.P., Li A., Yocum G.D., Robich R.M., Hayward S.A.L., Denlinger D.L. (2007). Up-Regulation of Heat Shock Proteins Is Essential for Cold Survival during Insect Diapause. Proc. Natl. Acad. Sci. USA.

[10] Wu G., Yi Y. (2018). Transcriptome Analysis of Differentially Expressed Genes Involved in Innate Immunity Following *Bacillus thuringiensis* Challenge in *Bombyx mori* Larvae. Mol. Immunol..

[11] Michaud M.R., Teets N.M., Peyton J.T., Blobner B.M., Denlinger D.L. (2011). Heat Shock Response to Hypoxia and Its Attenuation during Recovery in the Flesh Fly, *Sarcophaga crassipalpis*. J. Insect Physiol..

[12] Lah E.F.C., Musa R.N.A.R., Ming H.T. (2012). Effect of Germicidal UV-C Light(254 Nm) on Eggs and Adult of House Dustmites, *Dermatophagoides pteronyssinus* and *Dermatophagoides farinae* (Astigmata: Pyroglyhidae). Asian Pac. J. Trop. Bio..

[13] Kyre B.R., Rieske L.K. (2022). Using RNAi to Silence Heat Shock Protein Has Congeneric Effects in North America’s Dendroctonus Bark Beetles. Forest Ecol. Manag..

[14] Kyre B.R., Bentz B.J., Rieske L.K. (2020). Susceptibility of Mountain Pine Beetle (*Dendroctonus ponderosae* Hopkins) to Gene Silencing through RNAi Provides Potential as a Novel Management Tool. Forest. Ecol. Manag..

[15] Rodrigues T.B., Duan J.J., Palli S.R., Rieske L.K. (2018). Identification of Highly Effective Target Genes for RNAi-Mediated Control of Emerald Ash Borer, *Agrilus planipennis*. Sci. Rep..

[16] Chen X., Zhang Y. (2015). Identification of Multiple Small Heat-Shock Protein Genes in *Plutella xylostella* (L.) and Their Expression Profiles in Response to Abiotic Stresses. Cell Stress Chaperones.

[17] Xiong L., Liu Z., Shen L., Xie C., Ye M., Li Z., Zhang Z., Li J., Dong Y., You M. (2021). A Novel Reference for Bt-Resistance Mechanism in *Plutella Xylostella* Based on Analysis of the Midgut Transcriptomes. Insects.

[18] Zhang L.J., Wu Z.L., Wang K.F., Liu Q., Zhuang H.M., Wu G. (2015). Trade-off between Thermal Tolerance and Insecticide Resistance in *Plutella xylostella*. Ecol. Evol..

[19] Iwasaki S., Kobayashi M., Yoda M., Sakaguchi Y., Katsuma S., Suzuki T., Tomari Y. (2010). Hsc70/Hsp90 Chaperone Machinery Mediates ATP-Dependent RISC Loading of Small RNA Duplexes. Mol. Cell.

[20] Iwasaki S., Sasaki H.M., Sakaguchi Y., Suzuki T., Tadakuma H., Tomari Y. (2015). Defining Fundamental Steps in the Assembly of the *Drosophila* RNAi Enzyme Complex. Nature.

[21] Tsuboyama K., Tadakuma H., Tomari Y. (2018). Conformational Activation of Argonaute by Distinct yet Coordinated Actions of the Hsp70 and Hsp90 Chaperone Systems. Mol. Cell.

[22] Miyoshi T., Takeuchi A., Siomi H., Siomi M.C. (2010). A Direct Role for Hsp90 in Pre-RISC Formation in *Drosophila*. Nat. Struct. Mol. Biol..

[23] Cappucci U., Noro F., Casale A.M., Fanti L., Berloco M., Alagia A.A., Grassi L., Le Pera L., Piacentini L., Pimpinelli S. (2019). The Hsp70 Chaperone Is a Major Player in Stress-Induced Transposable Element Activation. Proc. Natl. Acad. Sci. USA.

[24] Iki T., Yoshikawa M., Nishikiori M., Jaudal M.C., Matsumoto-Yokoyama E., Mitsuhara I., Meshi T., Ishikawa M. (2010). In Vitro Assembly of Plant RNA-Induced Silencing Complexes Facilitated by Molecular Chaperone HSP90. Mol. Cell.

[25] Johnston M., Geoffroy M.C., Sobala A., Hay R., Hutvagner G. (2010). HSP90 Protein Stabilizes Unloaded Argonaute Complexes and Microscopic P-Bodies in Human Cells. Mol. Biol. Cell.

[26] Wang Y., Mercier R., Hobman T.C., LaPointe P. (2013). Regulation of RNA Interference by Hsp90 Is an Evolutionarily Conserved Process. Biochim. Biophys. Acta.

[27] Woehrer S.L., Aronica L., Suhren J.H., Busch C.J.-L., Noto T., Mochizuki K. (2015). A Tetrahymena Hsp90 Co-Chaperone Promotes siRNA Loading by ATP-Dependent and ATP-Independent Mechanisms. EMBO J..

[28] Zalucki M.P., Shabbir A., Silva R., Adamson D., Liu S.S., Furlong M.J. (2012). Estimating the Economic Cost of One of the World’s Major Insect Pests, *Plutella xylostella* (Lepidoptera: Plutellidae): Just How Long Is a Piece of String?. J. Econ. Entomol..

[29] Li Z., Feng X., Liu S.S., You M., Furlong M.J. (2016). Biology, Ecology, and Management of the Diamondback Moth in China. Annu. Rev. Entomol..

[30] Koch A., Kogel K. (2014). New Wind in the Sails: Improving the Agronomic Value of Crop Plants through RNAi-mediated Gene Silencing. Plant Biotechnol. J..

[31] Parker K.M., Barragán Borrero V., van Leeuwen D.M., Lever M.A., Mateescu B., Sander M. (2019). Environmental Fate of RNA Interference Pesticides: Adsorption and Degradation of Double-Stranded RNA Molecules in Agricultural Soils. Environ. Sci. Technol..

[32] Palli S.R. (2014). RNA Interference in Colorado Potato Beetle: Steps toward Development of dsRNA as a Commercial Insecticide. Curr. Opin. Insect Sci..

[33] Yang J., Chen S., Xu X., Lin S., Wu J., Lin G., Bai J., Song Q., You M., Xie M. (2023). Novel miR-108 and miR-234 Target Juvenile Hormone Esterase to Regulate the Response of *Plutella Xylostella* to Cry1Ac Protoxin. Ecotoxicol. Environ. Safe.

[34] Liu D., Asad M., Liao J., Chen J., Li J., Chu X., Pang S., Tariq M., Abbas A.N., Yang G. (2023). The Potential Role of the Piwi Gene in the Development and Reproduction of *Plutella xylostella*. Int. J. Mol. Sci..

[35] Jiang Y.-X., Chen J.-Z., Li M.-W., Zha B.-H., Huang P.-R., Chu X.-M., Chen J., Yang G. (2022). The Combination of *Bacillus thuringiensis* and Its Engineered Strain Expressing dsRNA Increases the Toxicity against *Plutella xylostella*. Int. J. Mol. Sci..

[36] Fu S., Liu Z., Chen J., Sun G., Jiang Y., Li M., Xiong L., Chen S., Zhou Y., Asad M. (2020). Silencing *Arginine Kinase*/*Integrin β* _1_
*Subunit* by Transgenic Plant Expressing dsRNA Inhibits the Development and Survival of *Plutella xylostella*. Pest. Manag. Sci..

[37] Palter K.B., Watanabe M., Stinson L., Mahowald A.P., Craig E.A. (1986). Expression and Localization of *Drosophila Melanogaster* Hsp70 Cognate Proteins. Mol. Cell. Biol..

[38] Lange B.M.H., Bachi A., Wilm M., González C. (2000). Hsp90 Is a Core Centrosomal Component and Is Required at Different Stages of the Centrosome Cycle in *Drosophila* and Vertebrates. Embo J..

[39] Tariq M., Nussbaumer U., Chen Y., Beisel C., Paro R. (2009). Trithorax Requires Hsp90 for Maintenance of Active Chromatin at Sites of Gene Expression. Proc. Natl. Acad. Sci. USA.

[40] Sarov M., Barz C., Jambor H., Hein M.Y., Schmied C., Suchold D., Stender B., Janosch S., KJ V.V., Krishnan R. (2016). A Genome-Wide Resource for the Analysis of Protein Localisation in *Drosophila*. eLife.

[41] Rosenzweig R., Nillegoda N.B., Mayer M.P., Bukau B. (2019). The Hsp70 Chaperone Network. Nat. Rev. Mol. Cell Bio..

[42] Schopf F.H., Biebl M.M., Buchner J. (2017). The HSP90 Chaperone Machinery. Nat. Rev. Mol. Cell Bio..

[43] Garbutt J.S., Reynolds S.E. (2012). Induction of RNA Interference Genes by Double-Stranded RNA; implications for Susceptibility to RNA Interference. Insect Biochem. Mol. Biol..

[44] Niu J.Z., Smagghe G., De Coninck D.I., Van Nieuwerburgh F., Deforce D., Meeus I. (2016). In Vivo Study of Dicer-2-Mediated Immune Response of the Small Interfering RNA Pathway upon Systemic Infections of Virulent and Avirulent Viruses in *Bombus terrestris*. Insect Biochem. Mol. Biol..

[45] Xie Y.F., Niu J.Z., Jiang X.Z., Yang W.J., Shen G.-M., Wei D., Smagghe G., Wang J.J. (2017). Influence of Various Stressors on the Expression of Core Genes of the Small Interfering RNA Pathway in the Oriental Fruit Fly, *Bactrocera dorsalis*. Insect Sci..

[46] Gupte T.M., Malik R.U., Sommese R.F., Ritt M., Sivaramakrishnan S. (2017). Priming GPCR Signaling through the Synergistic Effect of Two G Proteins. Proc. Natl. Acad. Sci. USA.

[47] Liu Z., Fu S., Ma X., Baxter S.W., Vasseur L., Xiong L., Huang Y., Yang G., You S., You M. (2020). Resistance to *Bacillus thuringiensis* Cry1Ac Toxin Requires Mutations in Two *Plutella xylostella* ATP-Binding Cassette Transporter Paralogs. PLoS Pathog..

[48] Buccitelli C., Selbach M. (2020). MRNAs, Proteins and the Emerging Principles of Gene Expression Control. Nat. Rev. Genet..

[49] Leu J.I.-J., Pimkina J., Frank A., Murphy M.E., George D.L. (2009). A Small Molecule Inhibitor of Inducible Heat Shock Protein 70. Mol. Cell.

[50] Kamal A., Thao L., Sensintaffar J., Zhang L., Boehm M.F., Fritz L.C., Burrows F.J. (2003). A High-Affinity Conformation of Hsp90 Confers Tumour Selectivity on Hsp90 Inhibitors. Nature.

[51] Yoon J.-S., Shukla J.N., Gong Z.J., Mogilicherla K., Palli S.R. (2016). RNA Interference in the Colorado Potato Beetle, *Leptinotarsa Decemlineata*: Identification of Key Contributors. Insect Biochem. Mol. Biol..

[52] De Wet J.R., Wood K.V., Helinski D.R., DeLuca M. (1985). Cloning of Firefly Luciferase cDNA and the Expression of Active Luciferase in *Escherichia coli*. Proc. Natl. Acad. Sci. USA.

[53] Li L.H., Zhu B., Sun X., Zheng K.W., Liang P., Gao X.W. (2023). miR-34-5p, a Novel Molecular Target against Lepidopteran Pests. J. Pest. Sci..

[54] Shen Z.J., Liu Y.J., Zhu F., Cai L.M., Liu X.M., Tian Z.-Q., Cheng J., Li Z., Liu X.-X. (2020). MicroRNA-277 Regulates Dopa Decarboxylase to Control Larval-Pupal and Pupal-Adult Metamorphosis of *Helicoverpa armigera*. Insect Biochem. Mol. Biol..

[55] García-Gomez B.I., Sánchez T.A., Cano S.N., do Nascimento N.A., Bravo A., Soberón M. (2023). Insect Chaperones Hsp70 and Hsp90 Cooperatively Enhance Toxicity of *Bacillus Thuringiensis* Cry1A Toxins and Counteract Insect Resistance. Front. Immunol..

[56] García-Gómez B.I., Cano S.N., Zagal E.E., Dantán-Gonzalez E., Bravo A., Soberón M. (2019). Insect Hsp90 Chaperone Assists *Bacillus thuringiensis* Cry Toxicity by Enhancing Protoxin Binding to the Receptor and by Protecting Protoxin from Gut Protease Degradation. mBio.

[57] Ono K., Eguchi T. (2023). Multiple Targeting of HSP Isoforms to Challenge Isoform Specificity and Compensatory Expression. Methods Mol. Biol..

[58] Fan Y., Song H., Abbas M., Wang Y., Li T., Ma E., Cooper A.M.W., Silver K., Zhu K.Y., Zhang J. (2021). A dsRNA-degrading Nuclease (dsRNase2) Limits RNAi Efficiency in the Asian Corn Borer (*Ostrinia furnacalis*). Insect Sci..

[59] Guan R., Chen Q., Li H., Hu S., Miao X., Wang G., Yang B. (2019). Knockout of the HaREase Gene Improves the Stability of dsRNA and Increases the Sensitivity of *Helicoverpa armigera* to *Bacillus thuringiensis* Toxin. Front. Physiol..

[60] Ludman M., Burgyán J., Fátyol K. (2017). CRISPR/Cas9 Mediated Inactivation of Argonaute 2 Reveals Its Differential Involvement in Antiviral Responses. Sci. Rep..

[61] You L., Bi H.-L., Wang Y.-H., Li X.-W., Chen X.-E., Li Z.-Q. (2019). CRISPR/Cas9-Based Mutation Reveals Argonaute 1 Is Essential for Pigmentation in *Ostrinia furnacalis*. Insect Sci..

[62] Zhu K.Y., Palli S.R. (2020). Mechanisms, Applications, and Challenges of Insect RNA Interference. Annu. Rev. Entomol..

[63] Xia J., Guo Z., Yang Z., Zhu X., Kang S., Yang X., Yang F., Wu Q., Wang S., Xie W. (2016). Proteomics-Based Identification of Midgut Proteins Correlated with Cry1Ac Resistance in *Plutella xylostella* (L.). Pestic. Biochem. Phys..

[64] You M.S., Yue Z., He W.Y., Yang X.H., Yang G., Xie M., Zhan D., Baxter S.W., Vasseur L., Gurr G.M. (2013). A Heterozygous Moth Genome Provides Insights into Herbivory and Detoxification. Nat. Genet..

[65] Ma X.L., He W.Y., Wang P., You M.S. (2019). Cell Lines from Diamondback Moth Exhibiting Differential Susceptibility to Baculovirus Infection and Expressing Midgut Genes. Insect Sci..

[66] Tang W., Yu L., He W., Yang G., Ke F., Baxter S.W., You S., Douglas C.J., You M. (2014). DBM-DB: The Diamondback Moth Genome Database. Database.

[67] Livak K.J., Schmittgen T.D. (2001). Analysis of Relative Gene Expression Data Using Real-Time Quantitative PCR and the 2^−ΔΔct^ Method. Methods.

