# Supplementary material for: Hsp70 and Hsp90 Elaborately Regulate RNAi Efficiency in Plutella xylostella
Source: Int J Mol Sci. 2023 Nov 10;24(22):16167. doi: 10.3390/ijms242216167 (PMC10671170; doi:10.3390/ijms242216167)
Supplement: Supplementary file 1 [file ijms-24-16167-s001.zip › ijms-2679116-supplementary.pdf]

Table S1. Sequences of primers used to amplify DNA fragments for gene cloning, dsRNA synthesis, qRT-PCR and vector construction.

**Primer**	**Sequence (5′-3′)**
PxHsc70-4-F	CTAGCTAATTCTTCGACTGA
PxHsc70-4-R	GCACAAAGAGAAGCATTAAG
PxHsp83-F	ATGCCTGAAGAAATGCAAGC
PxHsp83-R	TTAGTCGACTTCCTCCATGCG
dsPxHsc70-4-F	*TAATACGACTCACTATAGG*GAGAAGTCCACCAACAAGG
dsPxHsc70-4-R	*TAATACGACTCACTATAGG*TCGACCTCCTCGATGGTAG
dsPxHsp83-F	*TAATACGACTCACTATAGG*AGGAGGACAAGGTCAAGTT
dsPxHsp83-R	*TAATACGACTCACTATAGG*GGTCTCGTACAGCAGGAT
dsEGFP-F	*TAATACGACTCACTATAGG*GCACAAGTTCAGCGTGTCC
dsEGFP-R	*TAATACGACTCACTATAG*GGGGTGCTCAGGTAGTGGTT
dsPxmRPS18-F	*TAATACGACTCACTATAGG*ACCACTAGAGACAAGCATGAAGT
dsPxmRPS18-R	*TAATACGACTCACTATAGG*GATAGCCACCAGCAGTTCCT
dsLuc-F	*TAATACGACTCACTATAGG*GCCTATCATCCAGAAGATCATCA
dsLuc-R	*TAATACGACTCACTATAGG*GGCAGGTTAGACAGGTCGTA
dsRFP-F	*TAATACGACTCACTATAGG*AGACCATGAGAATCAAGGTGGT
dsRFP-R	*TAATACGACTCACTATAGG*TGTATGTGGTCTTGAAGTTGCAG
qPxHsc70-4-F	CTGCTGCTGCGATTGCTTA
qPxHsc70-4-R	CGGCGGTAGACTTGACTTC
qPxHsp83-F	ATCTTCCTCCGTGAGTTGAT
qPxHsp83-R	AGAGTTCCTTACCGCTGTC
qPxAgo2-F	TCTTCAAGCGACCGAACAAC
qPxAgo2-R	TGTGCGCCACATCAATGTTG
qPxDicer2-F	AAGCAATGCTTCCCGAACTG
qPxDicer2-R	AATTCTGCTGGCCGTAATGC
qPxmRPS18-F	CCGACTAAAGATCGCACGAAAG
qPxmRPS18-R	CCATTTCTGACGCATGACTTCC
qPxRPL32-F	CAATCAGGCCAATTTACCGC
qPxRPL32-R	CTGGGTTTACGCCAGTTACG
KpnI-PxHsc70-4-F	CGG**GGTACC**gccaccATGGCAACGAAAGCACCTG
EcoRI-PxHsc70-4-R	CCG**GAATTC**GTCGACCTCCTCGATGGTAG

Primer	Sequence (5′-3′)
KpnI-PxHsp83-F	CGG**GGTACC**gccaccATGCCTGAAGAAATGCAAGCG
EcoRI-PxHsp83-R	CCG**GAATTC**GTCGACTTCCTCCATGCGG
KpnI-Luc-F	CGG**GGTACC**gccaccATGGCCGATGCTAA
EcoRI-Luc-R	CCG**GAATTC**TTACACGGCGATCTTGCCGC
KpnI-PxAgo2-F	CGG**GGTACC**gccaccATGAAGATTAAACCAATGAAAATTCACC
EcoRI-PxAgo2-R	CG**GAATTC**CGAACGAAGAACATGGGGTTTCTC

Note: italic letters stand for the T7 promoter sequences; bold letters stand for restriction sites; lower-case letters stand for the Kozak sequences.

Table S2. *Hsc70-4* and *Hsp83* genes of insects for the phylogenetic analysis

**Species**	**Hsc70-4**	**Hsp83**
Leptinotarsa decemlineata	KC544268.1	XM_023160972.1
Tribolium castaneum	XM_961518.4	NM_001313877.1
Anoplophora glabripennis	XM_018720109.1	XM_018724339.1
Diabrotica virgifera virgifera	XM_028277987.1	
Melanoplus sanguinipes	KU218660.1	
Oxya chinensis		JQ859845.2
Locusta migratoria	AY299637.3	AY445913.3
Solenopsis invicta	XM_011170229.3	XM_011173022.3
Bombus terrestris	XM_003397414.2	XM_003396849.2
Apis mellifera	NM_001160050.1	NM_001160064.1
Anopheles gambiae	NM_079632.6	XM_308800.4
Musca domestica	NM_001309055.1	XM_005176875.3
Drosophila melanogaster	NM_079632.6	NM_079175.4
Plutella xylostella	NM_001305515.1	NM_001309114.1
Bombyx mori	NM_001043427.1	NM_001043411.1
Anticarsia gemmatalis	HQ223341.1	
Leguminivora glycinivorella		MK343468.1
Helicoverpa armigera	KX845567.1	GU230740.1
Mamestra brassicae	AB251896.1	
Ostrinia furnacalis		XM_028309301.1
Mythimna separata	MH669276.1	MF773751.1
Papilio polytes	NM_001311579.1	NM_001311546.1
Spodoptera frugiperda	MN480717.1	MN735781.1
Spodoptera litura		HM046609.1
Dendrolimus superans	EF194276.1	
Papilio memnon		GU230733.1

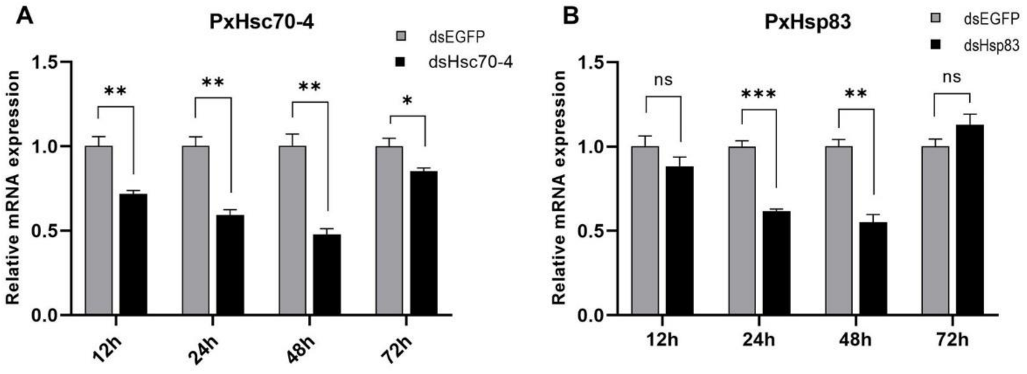

Figure S1. Effect of dsRNA transfection on the RNAi efficiency of *PxHsc70-4* and *PxHsp83*. (A) Effect of dsHsc70-4 transfection on the mRNA expression of *PxHsc70-4*. (B) Effect of dsHsp83 transfection on the mRNA expression of *PxHsp83*. DsEGFP was used as a control. Each experiment was repeated three times (mean ± SE, $n = 3$). Statistical significance was analyzed by multiple Student's t-test (ns, no significance; *, $p < 0.05$; **, $p < 0.01$; ***, $p < 0.001$).

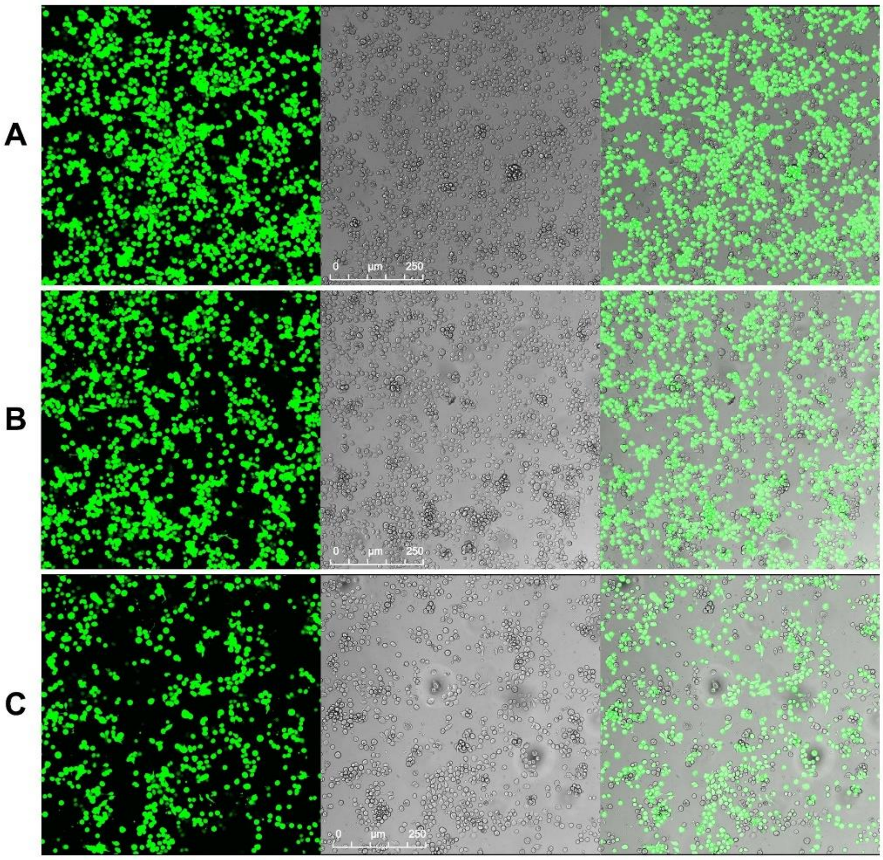

Figure S2. Green fluorescence expression of DBM cells transfected with the pIZT vectors. (A) pIZT vector; (B) pIZT-PxHsc70-4; (C) pIZT-PxHsp83. EGFP (left), brightfield (middle) and merge (right) were showed from left to right in the images. Scale bar of 50 μm was provided.

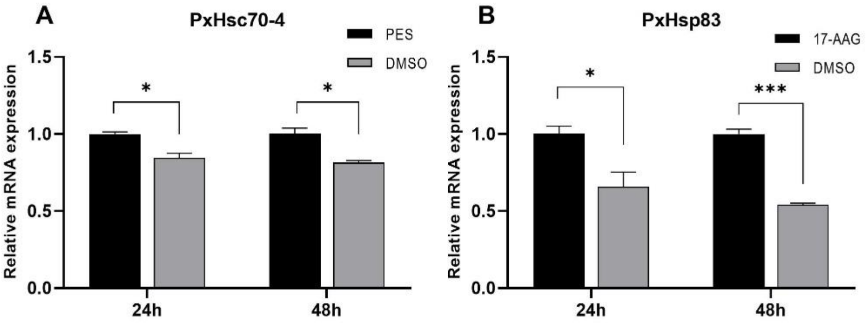

Figure S3. Effect of HSPs inhibition on the mRNA expressions of *PxHsc70-4* and *PxHsp83 in vitro*. (A) Effect of PES transfection on the mRNA expression of *PxHsc70-4*. (B) Effect of 17-AAG transfection on the mRNA expression of *PxHsp83*. DMSO was used as a control. Each experiment was repeated three times (mean ± SE, $n = 3$). Statistical significance was analyzed by multiple Student's t-test (*, $p < 0.05$; ***, $p < 0.001$).

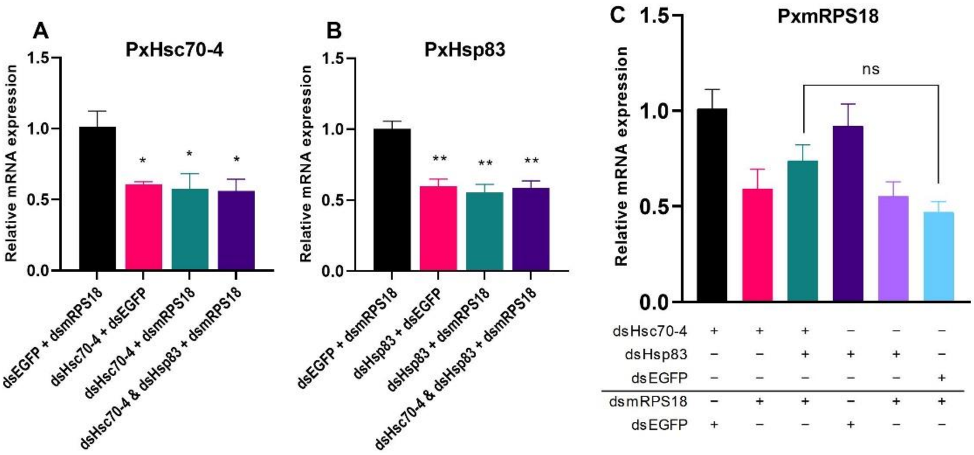

Figure S4. Effect of HSPs knockdown on the RNAi efficiency of *PxmRPS18 in vivo*. Effect of dsHSP and dsmRPS18 co-injection on the mRNA expression of *PxHsc70-4* (A), *PxHsp83* (B) and *PxmRPS18* (C) *in vivo*. DsEGFP was used as a control. Each experiment was repeated three times (mean ± SE, $n = 3$). Statistical significance was determined by one-way ANOVA with Tukey's test (ns, no significance; *, $p < 0.05$; **, $p < 0.01$).
